# Roles and mechanisms of tumour-infiltrating B cells in human cancer: a new force in immunotherapy

**DOI:** 10.1186/s40364-023-00460-1

**Published:** 2023-03-09

**Authors:** Enkui Zhang, Chengsheng Ding, Shuchun Li, Xueliang Zhou, Batuer Aikemu, Xiaodong Fan, Jing Sun, Minhua Zheng, Xiao Yang

**Affiliations:** 1 Department of General Surgery, Ruijin Hospital, Shanghai Jiao Tong University School of Medicine, Shanghai, 200025 China; 2 Shanghai Minimally Invasive Surgery Center, Ruijin Hospital, Shanghai Jiao Tong University School of Medicine, Shanghai, 200025 China; 3 Department of General Surgery & Carson International Cancer Research Center, Shenzhen University General Hospital and Shenzhen University Clinical Medical Academy, Shenzhen, 518055 China

**Keywords:** B cells, Cancer, Tumour microenvironment, Prediction, Immunotherapy

## Abstract

Immune checkpoint inhibitors (ICIs) targeting PD-1 or PD-L1 have emerged as a revolutionary treatment strategy for human cancer patients. However, as the response rate to ICI therapy varies widely among different types of tumours, we are beginning to gain insight into the mechanisms as well as biomarkers of therapeutic response and resistance. Numerous studies have highlighted the dominant role of cytotoxic T cells in determining the treatment response to ICIs. Empowered by recent technical advances, such as single-cell sequencing, tumour-infiltrating B cells have been identified as a key regulator in several solid tumours by affecting tumour progression and the response to ICIs. In the current review, we summarized recent advances regarding the role and underlying mechanisms of B cells in human cancer and therapy. Some studies have shown that B-cell abundance in cancer is positively associated with favourable clinical outcomes, while others have indicated that they are tumour-promoting, implying that the biological function of B cells is a complex landscape. The molecular mechanisms involved multiple aspects of the functions of B cells, including the activation of CD8+ T cells, the secretion of antibodies and cytokines, and the facilitation of the antigen presentation process. In addition, other crucial mechanisms, such as the functions of regulatory B cells (Bregs) and plasma cells, are discussed. Here, by summarizing the advances and dilemmas of recent studies, we depicted the current landscape of B cells in cancers and paved the way for future research in this field.

**Graphical Abstract:**

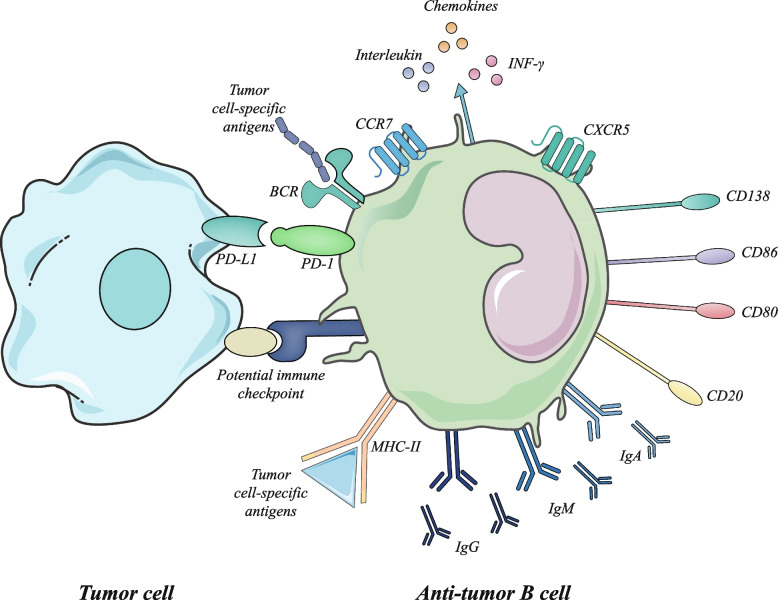

## Introduction

Immune checkpoint inhibitor (ICI) therapy has now been established as a first-line treatment for multiple types of metastatic cancers. Many of these promising therapeutic strategies, which can lead to strong antitumour responses and even long-term tumour remission, are based on the blockade of the activation of immune inhibitory receptors such as programmed cell death protein 1 (PD-1), programmed cell death ligand 1 (PD-L1) and cytotoxic T lymphocyte–associated protein 4 (CTLA-4) [1, 2]. Unfortunately, only a minority of patients showed satisfactory responses to ICIs. Therefore, it is urgent to expand the understanding of the potential mechanisms leading to resistance to ICI therapy [3].

The tumour microenvironment (TME) is the environment in which tumours form and develop. It consists of stromal cells (including mesenchymal cells and endothelial cells), extracellular matrix, various cytokines and chemokines, and most importantly, various components of immune cells [4]. Accumulating evidence has suggested that the interaction between cancer cells and the TME reshapes the immune phenotype of cancers and thereby has profound effects on cancer initiation, progression, and therapeutic efficacy [5]. TME-mediated ICI resistance can be induced by cytokines secreted by tumour or stromal cells [6, 7]. Additionally, the interaction of tumour cells with stromal cells or components of the extracellular matrix can also blunt the therapeutic response [2]. Combinations of therapies targeting TME components and chemotherapy, radiation, or ICIs now appear to offer new approaches for cancer treatment. Driven by recently developed techniques such as single-cell sequencing and spatial transcriptomics, new subsets of immune cells in the TME have been defined and located [8, 9].

Tumour-infiltrating B cells are often colocalized with T cells or other immune cells, such as dendritic cells, in organized lymphoid aggregates known as tertiary lymphoid structures (TLSs). Substantial evidence suggests that the response to ICIs is positively correlated with the presence of B cells, particularly in TLSs [10]. Mechanistically, B cells secrete an array of cytokines (including TNF, interleukin (IL)-2, IL-6 and IFN-γ), through which they recruit other immune effector cells, including T cells [11, 12]. Memory B cells may act as antigen-presenting cells, driving the expansion of both memory and naive tumour-associated T-cell responses, and potentially contribute to the antitumour response by producing antibodies against tumours [13]. Moreover, B cells probably cooperate with the other key immune contexture within the TLS. However, recent studies have reported that B cells dampen the tumour immune response by producing certain cytokines, such as IL-10, IL-35, TGF-β and even gamma-aminobutyric acid (GABA) [14]. All these results indicate that B cells have heterogeneous subpopulations with distinct functions, contributing to pros as well as cons in antitumour immune responses. In this review, we summarized and updated recent findings pertaining to the classifications, functions, and mechanisms of B cells in cellular and humoral immune responses to cancer and their potential relevance to immunotherapy.

## Phenotypes and functions of B cells in the TME

### B-cell development and recruitment

B cells are derived from the bone marrow and then circulate and move to the spleen and lymph nodes. They participate in immune responses in the peripheral blood and immune zone. Naive B cells are activated in response to specific antigens during development and then differentiate and proliferate into plasma blasts or plasma cells (PCs), which are known as activated B cells. Depending on the function of differentiation, other B cells become memory B cells, follicular B cells or regulatory B cells (Bregs) [15, 16]. The epitopes of B cells change with the transformation and maturation of B cells, and the method of labelling B cells depends on the cluster of differentiation (CD), such as the well-known CD19, CD20, CD21, CD40, and CD79b. Furthermore, single-cell RNA sequencing (scRNA-seq) can be used to analyse the distributions and types of B cells in tissues, which is especially applicable to the heterogeneity of B cells in tumours [8]. We can define a richer B-cell phenotype to reveal the key mechanisms of tumour immunology.

B cells mainly exist in the form of a scattered distribution or TLSs in the TME and directly and indirectly exert effects on tumour cells through antigen presentation, antibody production or cytokine production. According to previous concepts, the infiltration of B cells in the TME can also be divided into the immune inflammatory type, immune excluded type and immune desert type [17]. The factors that influence B-cell recruitment are not fully understood. What is clear is that in addition to receiving antigen stimulation and BCR signalling, many cytokines are also involved in the regulation of the B-cell population and status. For example, IL-17 and CXCL12 can induce pro-B progenitor cells to grow into B cells and participate in the evolution of B cells [18]. INF-α, -β, and -γ produced by other immune cells, such as T cells, can also promote B-cell activation and plasma cell differentiation to produce antibodies [19, 20]. B cells are recruited to the mouse intestinal epithelium in response to CCL20 and CCL28 [21]. Moreover, B cells can transform into antigen-presenting cells (APCs) through the membrane-binding molecule CD40/CD40L induced by IL-4 and IL-21 [22, 23]. In the TME, the cytokine recruitment of B cells represented by CXCL13 is different from that in the normal immune environment [24].

### APC

Acting as professional APCs is the crucial function of B cells in the TME. Compared with other APCs, antigens are recognized more sensitively by the BCR [25]. B cells enhance the density and response of T cells in the TME by activating T cells, especially CD4+ T cells [26]. On the one hand, B cells transform CD4+ T cells into an activated state, which enhances the density of T cells in the TME. On the other hand, B cells mediate the transformation of CD4+ T cells and CD8+ T cells to different functional subsets after receiving antigen stimulation and then strengthen the specific response of T cells [27]. In addition, B cells tend to be actively converted into effector B cells. Some subsets of B cells express surface markers, including MHCII, CD80 and CD86, and can maintain additional T-cell population expansion after dendritic cell (DC)-induced initial activation by acting as APCs [28]. B cells also affect other APCs and enhance their ability to present antigens, such as in DCs and macrophage antigen presentation. B cells affect the state of both and develop in the direction of promoting immunity [29]. The mechanism of B-cell recognition of cancer cell antigens is the focus of current research in B-cell treatment.

### “Helper” of T cells

Elimination of B cells abrogates the activation of cytotoxic and helper T cells (CTL and Th) upon antigen stimulation, indicating that antigen-specific interactions between T cells and CD20+ B cells in the TME seem to be crucial to the protective role of T cells [30]. Intriguingly, activated CTLs can also engage with soluble CD27 secreted by CD19+ B cells, which promotes their survival and proliferation, suggesting a ‘helper’ role of B cells in the tumour immune response [31].

In several recent studies on TLSs in cancer, TLSs have been found to be strongly associated with a favourable prognosis. B cells, as the major component in TLSs, contribute significantly to T-cell immunity. Two major functions of TLSs are the mediation of T cells to enhance antitumour effects and the crosstalk of T and B cells to promote the maturation and development of TLSs. Corresponding to the abovementioned enhanced immunity to fight tumours is the anti-immune regulatory function of Bregs. Bregs suppress the immune activity of T cells and other immune cells by secreting related cytokines such as IL-10 and IL-35 [32]. B cells from the TME have the ability to play distinct roles by differentiating groups to affect the immune balance of the tumour.

### The production of immune substances

B cells regulate immune function and the immune microenvironment by producing immune substances, including antibodies and cytokines. For cytokines produced by B cells, it has been clearly confirmed that IL-2 and IL-4 produced by B cells can induce Th cells to differentiate into Th1 and Th2 cells [33, 34], and IL-6 can induce the activation of regulatory T cells (Tregs) and promote the production of plasma cell antibodies [35]. B cells can produce IFN-α and IFN-γ to activate the cell-killing effect of NK cells [36]. In addition, Bregs produce cytokines to suppress immune function, including the secretion of IL-10, IL-35, and TGF-β by Bregs to inhibit T cell, DC, and macrophage function. In addition, Bregs can also exert inhibitory effects by the membrane-bound molecules CD39, CD73 and PD1 [37]. A higher proportion of Bregs is often found in advanced hepatocellular cancer (HCC), gastric cancer and prostate cancer, indicating that Bregs may influence tumour development and progression [38–40]. Moreover, Bregs also secrete cytokines such as TGF-β, which may differentiate naive CD4+ T cells into Tregs and transform macrophages into an M2 immunosuppressive phenotype, leading to remodelling of the TME [41, 42].

Antibody production is a specific ability of B cells, and increasing research points to the role of antibodies in tumours. During tumorigenesis, frequent missense mutations or alternative splicing events often result in the exposure of tumour-specific neoantigens that can drive the B-cell-mediated humoral immune response. With B-cell maturation confirmed, they perform clonal proliferation, selection for high-affinity antibodies and isotype switching within tumour-associated TLSs and in less organized structures, ultimately converting to effector or memory B cells and PCs [16, 43]. Furthermore, PCs and memory B cells themselves produce high titres of tumour-specific antibodies, inducing opsonization, complement-mediated lysis of cancer cells, antibody-dependent cell cytotoxicity (ADCC) executed by T cells or NK cells and antibody-mediated phagocytosis of tumour cells by macrophages in TLSs of the TME [10, 28]. Moreover, the IgG-type antibodies secreted by PCs can be directed to tumour-associated antigens, induce ADCC in bound tumour cells and enhance the complement pathway through FcγR activation, reflecting the role of B cells as the 'defender' [44–47]. These results confirm that B cells produce an emerging antitumour response that increases with TLSs and the TME.

## Crosstalk of B cells with other immune cells in the TME

### Obvious antitumour effects

Both B cells and other immune cell subsets in the TME interact with each other through multiple pathways to influence oncogenesis and might determine the response to immunotherapy. Substantial evidence suggests that B cells are the main partners of T cells in antitumour immunity. B-cell and T-cell aggregation in the TME has traditionally been considered a favourable prognostic feature, which has also been validated using single-cell techniques [48]. B cells can provide T cells with stronger signals, such as the inducible costimulator (ICOS) ligands CD80 and CD86, to support the function of T cells in immune killing [49, 50]. The aggregation of CD20+ B cells and CD8+ T cells plays a crucial role in the tumour immune response through costimulatory signalling, such as CD40/CD40L, which triggers the tumour-killing effect of T cells [51]. Different subsets of T cells are recruited into the TME to exert antitumour effects by the secretion of cytokines from B cells. CD20+ B cells recruit CD8+ T cells by releasing chemokines, and then B-cell populations attract T cells in inflammatory responses and immune cell interactions, including common chemokines such as CCL3, CCL4, CCL5, CXCL10, and CXCL13. In particular, CXCL13 is a core chemokine that recruits tumour-infiltrating B and T cells [52–54].

Correspondingly, helper T cells also prompt B cells to differentiate PCs and switch antibodies that exhibit antitumour responses [55]. Another recent research advance points to T follicular helper (Tfh) cells. Interactions between CD4+ T cells and B cells within tumours induce tumour-specific Tfh cell generation; afterwards, Tfh cells can enhance the antitumour effect of CD8+ T cells by secreting IL-21 [27] In addition, CD86+ B cells induce T-cell responses by localizing heavily in the TME through the presentation of tumour-associated antigens, particularly in TLSs of various cancer types, and B cells have also been found to promote the formation of TLSs by secreting CXCL13 and cytotoxic factors [28]. These results indicate that B cells in the TME achieve antitumour immunity by activating and inducing various T-cell subsets.

**Fig. 1:**
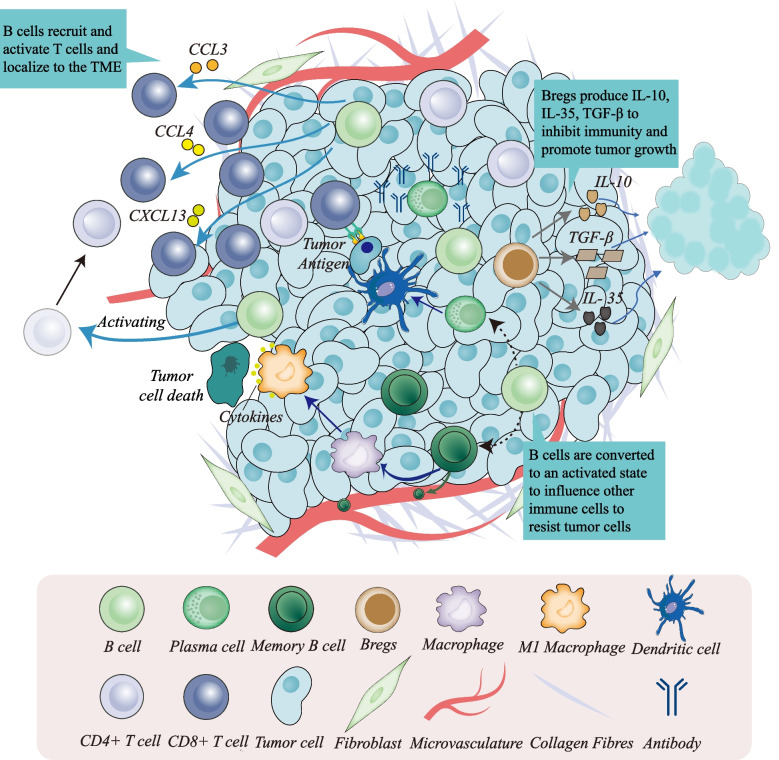
Crosstalk of B cells with other immune cells in the tumour microenvironment. Tumour-infiltrating B cells can activate CD4+ T cells, recruit CD8+ T cells and regulate the polarization of macrophages. In addition, B cells transform into plasma cells and effector B cells in the TME. Plasma cells can influence dendritic cells to present antigens to CD8+ T cells and produce antibodies to inhibit tumours. Plasma cells secrete antibodies to combine with the ligand of macrophages and transform macrophages into the M1 type to kill tumour cells. Effector B cells travel into the blood circulation to defend against potential tumour cells. However, Bregs can produce cytokines that are immunosuppressive and ultimately promote cancer development

Crosstalk between T cells and B cells in the immune microenvironment is the backbone of tumour immunity, and other immune components also closely contact B cells. B cells are converted from naive B cells to memory B cells and PCs in TLSs of the TME [56]. Memory B cells have been detected in the peripheral blood of patients with various tumours, which may be related to the role of B cells in the microcirculation to help fight metastatic tumour cells [44, 57, 58]. Furthermore, PCs express IgA, IL-10, and PD-L1, resulting in immunosuppressive effects [59]. Recent studies have also shown the potential ability of B cells located in TLSs to respond to immunotherapy in multiple cancers [28, 60, 61]. With the help of Tfh cells, memory B cells and PCs are generated during the maturation of TLSs [62]. IgG-secreting PCs can also enhance the immune responses of Th1/Th17 cells [63]. A fraction of DCs expressing FcγR have affinity for PC-secreted IgG and enhance the immune response. Therefore, the utilization of the affinity of tumour-specific IgG antibodies to further improve the effect of antibody therapy for cancer is one of the current research directions in cancer immunotherapy [64, 65]. Effector B cells can affect the migration of DCs and produce antibodies to induce M1 macrophages to undergo ADCC or antibody-dependent cellular phagocytosis (ADCP) to kill tumour cells [66]. In addition, B cells crosstalk with other cells in the TME. Fibroblasts from tumours express higher levels of CXCL13, BAFF, and APRIL and are dependent on the CXCR5-CXCL13 axis to promote the accumulation of B cells in tumours and increase the proliferation of fibroblasts and TLS expansion in the TME [67]. These results suggest that B cells positively affect the TME and antitumour responses through the combined action of B cells and multiple immune cells (Fig. 1).

### Hidden protumor efforts

Overall, the antitumour effects of B cells are indisputable, but in fact, not all B cells have antitumour effects, and Bregs are the first to bear the brunt. Bregs negatively regulate the immune response by producing anti-inflammatory factors such as IL-10, IL-35 and TGF-β, which has been confirmed in several related tumour studies [32]. In the immunomodulation of pancreatic cancer, Bregs cause the depletion of CD8+ T cells in the TME through the IL-35- and IL-10-mediated STST2 pathway [68]. Bregs have been detected in the peripheral blood of gastric cancer patients and mediate tumour cell escape through IL-10 signalling [69]. In a clinical study of chronic myeloid leukaemia, tumour-produced TGF-β induced an immunosuppressive phenotype in Bregs, resulting in accelerated tumour progression [70]. These results are predictable considering the immunoregulatory function of Bregs, and reasonable elimination of the tumour-promoting effect of Bregs is the main point of current attention.

On the other hand, it is the tumour-promoting effect of antibodies produced by PCs, mainly including IgG variants and IgA. IgG4 subclass antibodies have been shown to be highly correlated with tumour malignancy and poor prognosis in oesophageal cancer patients, and the same results have been found in mouse models of breast cancer, colorectal cancer and carcinogen-induced skin papilloma [71]. In a colorectal cancer study, IgG4 induced the activation of M2 macrophages, impairing anticancer effects and promoting tumour development [72]. Regarding the tumour-promoting effect of IgA, it has been found that the concentration of IgA is positively correlated with a poor prognosis in both melanoma and HCC [73, 74]; it may regulate the immune function of T cells and cause a decrease in antitumour ability. These studies roughly suggest the opposite roles of antitumour immunity in human tumours and mouse tumours. In some animal experiments in mice, as ‘promoters’, B cells mostly promote tumour survival through specific cytokines. The conclusions of this part also require continuous attention and research.

## B cells as biomarkers in cancer

### Prognostic markers in cancer

Multiple phenotypes of B cells have been shown to infiltrate various tumour tissues and are associated with a favourable prognosis (Table 1). These B cells perform complex functions in the TME. On the one hand, they can activate and connect with other immune cells to enhance the killing of tumour cells; on the other hand, different types of B cells produce various cytokines and antibodies and remodel the TME. The following characteristics of B cells as biomarkers can generally be found in patients with tumours: (i) The main types of infiltrating B cells are naive B cells, PCs and memory B cells, and the number of infiltrating B cells in tumours is positively correlated with the prognosis of patients. To a certain extent, the heterogeneity of populations and types of B cells in tumours determines the degree of antitumour effect. (ii) The spatiotemporal heterogeneity of B cells in tumours also affects the prognosis of cancer. Patients with advanced tumours have a low number of effector B cells and a high proportion of naive B cells. In tumour tissues, PCs and memory B cells are more relevant than naive B cells for the positive prognosis of tumours. In peripheral blood, there is also some degree of variation in the composition of B cells. (iii) TLSs are an important form of tumour-infiltrating B cells. The presence or absence of TLSs, the number of TLSs in the tumour and the degree of TLS maturation have been shown to be valuable prognostic markers. (iv) Antibodies produced by PCs can also be used as a prognostic indicator.

**Table 1:** Prognosis and prediction of response to immunotherapy in human cancer

**Tumour type**	**Cell type**	**Sample number**	**Cell marker**	**Location of B cells**	**Clinical findings**	**Reference**
**Melanoma**	B cells	136	CD20	Peritumoral TLS	Better response to ICIs	[75]
**Melanoma**	B cells	164	CD20	TLS	Improved OS	[58]
**Sarcoma**	B cells	496	CD20/CD20	TLS	Better response to ICIs	[76]
**Breast cancer**	B cells	1200	CD20	Intratumoural tissue	Improved DSS	[77]
**Breast cancer (HER2+)**	B cells	136	CD20	Intratumoural tissue	Prolonged OS and DFS	[78]
**Breast cancer (TNBC)**	B cells	113	CD20	Intratumoural tissue	Prolonged OS and DFS	[78]
**Breast cancer (TNBC)**	B cells	114	CD20/CD138	Intratumoural tissue	Improved DFS	[79]
**Colorectal cancer**	B cells	316	CD20	stromal	Improved DSS	[80]
**Colorectal cancer**	B cells	557	CD20/CD138	Intratumoural tissue	Improved OS	[81]
**Gastric cancer**	B cells	266	CD20	TLS	Improved OS	[82]
**Gastric cancer**	Bregs	59	CD19	/	Poorer OS	[39]
**Hepatocellular carcinoma**	B cells	120	CD20	Intratumoural tissue	Prolonged OS	[83]
**Hepatocellular carcinoma**	B cells	112	CD19/CD20	Intratumoural tissue	Improved survival rate	[84]
**Pancreatic cancer**	B cells	28	CD20	TLS	Improved OS	[56]
**NSCLC**	B cells	196	CD20	TLS	Improved DSS	[44]
**NSCLC**	PCs	207	CD20/CD138	Intratumoural tissue	Prolonged OS	[85]
**Renal cell cancer**	PCs	130	IgG+/IgA+	TLS	Prolonged PFS	[46]
**Prostate cancer**	B cells	110	CD20	Intratumoural tissue	Poorer RFS	[59]

Some tumour-infiltrating B cells secrete antibodies against breast cancer antigens to promote humoral immunity. IgG and IgA are the primary antibodies, and they show different responses, with IgG usually representing a positive prognosis and IgA representing the opposite [72]. By initiating self-activation, B cells achieve class switching and affinity maturation in the immune aggregation zone and act as 'helpers' and 'defenders', thereby expanding the antibody library that matches adaptive immunity [16, 62]. In vitro studies have demonstrated that B cells from melanoma patients can produce IgG, which can eliminate cancer cells by triggering ADCC and ADCP. IgG can enhance antigen presentation and phagocytosis to promote antitumour effects [66]. Variants of IgG have also been detected in the peripheral blood of patients with non-small cell lung cancer (NSCLC) and breast cancer (BC), which is usually associated with a better clinical prognosis. In contrast, IgA has been detected in the TME and blood and has been associated with tumour-promoting mechanisms in some animal experiments [86, 87]. For example, TGF-β and some interleukins can induce IgA production, resulting in immunosuppression [59]. Consistently, tumour-specific antibodies have been detected in the serum of cancer patients in many studies [88]. Furthermore, the detection of serum levels of antibodies against tumour-associated antigens has been proposed for the early detection of cancer. In addition, the role of BCR in immunotherapy should not be overlooked. The killing effect of BCR and some costimulatory molecules, such as CD40 and CD86, on the surface of B cells in response to external antigen stimulation or enhancement of costimulatory signals of CD4+ T cells in tumours can also be used as markers for clinical prognostic monitoring [28, 89, 90].

Several recent heavyweight studies have shown that B-cell localization in the TME is a predictor of a favourable response in patients treated with ICIs in melanoma, sarcoma and clear cell renal cell carcinoma. Multicolour fluorescence immunohistochemical staining of some tumour tissues also revealed that there are many different lineages of B cells localized in TLSs, such as CD19, CD20, CD21, and CD138 [46, 58, 76]. Different B-cell lineages and tumour backgrounds have also shown different roles in various cancer types.

### The role of B cells in immunotherapy and chemoradiotherapy

After changing the ecology of tumour immunity, B cells also respond positively to immunotherapy. This finding has been confirmed to varying degrees in different cancers. In addition to the involvement of B cells in tumour immune responses and influencing patient prognosis, B cells can also be used to predict the response to immunotherapy. From the classic classification, activated B cells, represented by PCs and memory B cells, enriched in tumour tissue usually predicts a favourable prognosis [91]. They adjust T-cell states to make them more sensitive to tumour cells in the TME. In melanoma patients receiving CTLA-4 inhibitor therapy, a lack of B cells is a predictor of a poor response to ICIs [92]. The signatures of B cells and PCs show a high immune enrichment status and a strong response to anti-PD-1 antibody therapy in soft tissue sarcoma [76]. In the immunotherapy of breast cancer, B-cell activation by T follicular helper cells and the secretion of antibodies is a key reflection of the response to immunotherapy. The response can also be checked by screening antibodies [93]. CD20+ B lymphocytes in colorectal cancer (CRC) are correlated with other immune cells and support a beneficial prognostic impact, even as prognostic biomarkers in metastatic CRC. Related B-cell subtypes are becoming hot targets for research [80, 94]. In addition, B cells can be used as biomarkers for immunotherapy, and B-cell-based immunotherapeutic strategies are discussed, including the stimulation of B-cell ligands to enhance B-cell immunity and activation of B cells in vitro and in vivo [95].

It is noteworthy that the characteristics of B cells in the TME change after ICI treatment, which has been demonstrated in different cancers (Table 2). After ICI therapy, responders were found to have the following characteristics: (1) B cells were significantly more abundant in the TME of responders than nonresponders, and the types were mainly memory B cells and plasma cells [46, 75, 85, 96–98]. (2) A variety of specific BCRs and antibodies closely related to B cells were also found to be abundant in the TME, with the type of antibodies being mainly immune-promoting IgG [46, 75, 93, 96]. (3) Increasing numbers of memory B cells, antibodies and cytokines were also detected in the peripheral blood of responders [98, 99]. Thus, these results confirmed not only the strong association between altered B-cell characteristics in the TME and the response to ICI treatment but also that B cells are likely to be necessary players in the role of ICIs in the TME.

**Table 2:** Changes in B cells in the TME after ICI treatment in responders

**Cancer type**	**ICI**	**Number of patients**	**Changes in B cells in the TME of responders**	**Other increasing biomarkers**	**Reference**
**Melanoma**	Nivolumab, Ipilimumab	41	High proportion of class-switched memory B cells, activated B cells and GC-like B cells	BCR types, B-cell RNA signatures	[75]
**Melanoma**	Nivolumab, Ipilimumab	64	Higher memory B cells and naive B cells	Clonal BCR repertoire, IgG	[96]
**RCC**	Nivolumab, Ipilimumab	59	Higher plasma cells	IgG	[46]
**NSCLC**	Atezolizumab	344	Higher B cells and plasma cells	TLSs	[85]
**NSCLC**	Anti-PD-1 treatment	12	CD20+CD22+ADAM28+ B cells in TLSs	-	[100]
**NSCLC**	Nivolumab	150	-	IgM+ memory B cells in peripheral blood	[99]
**Cervical cancer**	Anti-PD-1 treatment	8	Higher B cells	Higher expression of PD-L1	[97]
**HCC**	Cabozantinib combined with Nivolumab	15	Higher B cells, CD138+ B cells	Higher TNF-α expression and TLSs	[98]
**SCC (Mouse model)**	Anti-PD-L1 treatment	-	B cells	IgM, IgG	[101]
**BC (Mouse model)**	Anti-PD-1 and CTLA-4 treatment	-	Class-switched plasma cells	IgG	[93]

For some patients receiving chemotherapy, there was an increase in the number of B cells, especially naive B cells and PCs, in patients who responded to treatment. These patients eventually had improved disease-free survival (DFS) and overall survival (OS). The number of B cells can be used as a prognostic indicator for cancer patients after radiotherapy or chemotherapy [101–103]. In other studies, dabrafenib plus trametinib in melanoma patients increased the density of tumour-infiltrating B cells in responders and may induce TLS formation [104]. The phenotype of tumour-infiltrating B cells changed to ICOSL+ B cells after chemotherapy in breast cancer patients and was associated with an improved prognosis. A positive correlation was also found between the response to chemotherapy in mice and the B cells infiltrating the tumour [105]. In ovarian cancer patients who responded to neoadjuvant chemotherapy, the density of CD20+ B cells was increased [106]. These results suggest that the density of B cells is closely related to the chemotherapy response. The number of B cells and TLSs in tumours can be increased with the addition of chemotherapy to enhance the immune response and improve tumour treatment by synergistic immunotherapy.

## The future direction of B-cell clinical research

**Fig. 2:**
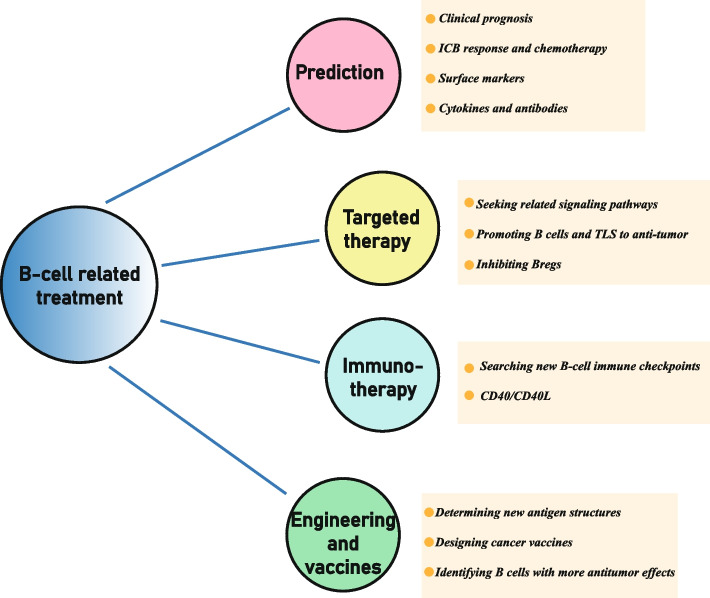
The current mainstream research direction in B-cell treatment. It encompasses four main categories, including cancer prediction, targeted therapy, immunotherapy, B-cell engineering and related vaccine research

As the only immune cells that can produce antibodies, B cells can be expected to become a force in immunotherapy and targeted therapy. At present, the clinical application of cancer-targeting B cells is being attempted. We focus on these studies and summarize current advances (Fig. 2).

### Predictive role

Prognostic prediction and treatment evaluation based on the surface markers of B cells have made distinct progress in B-cell clinical research at present. CD19, CD20, CD38, CD86 and CD138 detected in tumour tissues were used as markers for positive prognosis [28, 58, 84, 85]. These markers mainly correspond to naive B cells, switch B cells, PCs and memory B cells. In addition, the presence of B cells in tumours, especially fully mature TLSs, also plays a crucial role in cancer prediction. B cells can better exert antitumour effects by producing antitumour antibodies and cooperating with other immune cells in mature TLSs [61, 107]. The changes in IgG, IgM, and IgA produced by B cells in tumour tissues or peripheral blood can also reflect the prognosis of cancer. Moreover, the detection of Bregs is important for predicting cancer. The corresponding markers were mainly IL-10, IL-35 and PD-L1. In addition, some B-cell markers sometimes correspond to a poorer prognosis, such as CD19 (which was found to be associated with a poor prognosis in studies of breast and gastric cancers) and CD79a expression on immature myeloid cells, contributing to their tumour-promoting effects [39, 108, 109]. This suggests that the link between these B-cell biomarkers and prognosis remains a focus of research. B cells are also good predictors of the response to immunotherapy and chemoradiotherapy. In addition to the above biomarkers, targeting the B-cell transcriptome can be used to construct effective prognostic models for cancer patients.

### Targeted therapy

It is valuable to look for antitumour or tumour-promoting signals of cytokines and antibodies associated with B cells to find some targets for targeted therapy. Cytokine-based signalling pathways, such as the CXCL13/CXCR5 axis and the CCL19,21/CCR7 axis, mediate the recruitment of B cells and TLSs in tumours [62]. CXCR5 is the target molecule of CXCL13, which induces the aggregation of CXCR5-expressing B cells and other immune cells in the TME, and it is also highly correlated with the expression and formation of TLSs. CCL19,21/CCR7 can mediate the homing of immune cells to lymphoid tissues, leading to enhanced immune infiltration in the TME and ultimately to a positive survival outcome. In addition, cytokines such as IL-17 and CXCL12 can contribute to the maturation of B cells [110]. These results suggest that B-cell-related cytokines and pathways are available as targets for tumour-targeted therapy to inhibit tumour growth. The main goal is to enhance the infiltration of B cells or TLSs in the TME and obtain a better immune benefit. However, the above cytokines also showed opposite results in some studies. For instance, CXCL13 elevation was related to a poor prognosis in breast cancer, and CCR7 upregulation in some cancer cells enhanced lymph node metastasis [111–113]. This may be closely related to the heterogeneity of different tumour types. It is important to note that when enhancing B-cell-based immunity, attention needs to be paid to its hidden protumor effects as well as the massive production of antibodies and cytokines causing immune storms and autoimmune diseases. In contrast to the antitumour effects of B cells, Bregs are also a potential target, and Breg-produced IL-10 and IL-35 can have inhibitory effects on a variety of immune cells promoting tumour development. This demonstrates that tumour development can be inhibited in tumours by suppressing Breg-secreted cytokines.

### Immunotherapy

B cells in the TME of patients who have responded to ICI treatment tend to be more active. This state is mainly characterized by differentiation towards specific functional subpopulations and possibly increased expression of immune checkpoints [114, 115]. Therefore, the identification of developmental trajectories and specific subpopulations of tumour-infiltrating B cells using single-cell technology is an important future direction [116, 117]. On the one hand, a better prognosis could be achieved by promoting the differentiation of B cells into specific subpopulations. On the other hand, novel immunotherapeutic drugs could be designed based on new surface markers and potential immune checkpoints of B cells.

Recently, CD40/CD40L was identified as a potential immune checkpoint for B cells and may play a role in immunotherapy [118]. CD40 agonist antibodies have been shown to make a difference in the clinical treatment of different cancers and are closely related to B cells [119]. CD40 expressed by B cells generates signalling to stimulate the formation of immune tissue generative centres, promotes antibody isotype switching and enhances antigen affinity, and then creates long-lasting PCs and memory B cells [120]. Targeting CD40/CD40L is still in clinical trials in some solid tumours, but it gives us the idea to further search for B-cell immune checkpoints for immunotherapy, which is an important research direction for B-cell immunotherapy.

### B-cell engineering and vaccines

A number of studies are underway using CAR-T and cancer vaccines as important tools for cell engineering to treat cancer [121, 122]. However, B cells and their interactions with T cells have been much less studied in cancer cell engineering and vaccines than T cells. Although B cells are highly sensitive to tumour cell-specific antigens, these antigens require antigen-antibody interactions mediated by CD4+ T cells with the help of MHC-II molecules. Subsequently, B cells are triggered to produce antibodies upon contact with tumour cell-specific antigens and completely destroy the target antigen. This requires determining the structure and form of the processed antigen of the B-cell-recognized MHC molecule as the basis for in vitro processing and loading and applying it in the form of a vaccine to cancer patients to induce adaptive immunity in B cells [123]. Current single-cell omics technology is a reliable means of classifying subpopulations of tumour-infiltrating B cells and identifying B cells with better antitumour effects.

## Discussion

Studies on immune cells in tumorigenesis and development raise concern and indicate the need for more effective immune treatments. B cells are gaining attention as an emerging prognostic factor and immunotherapy direction. We discuss that different subsets of B cells exhibit different immune functions against cancer. In the TME, B cells interact with other immune cells in a variety of ways in different states, mainly by inhibiting the occurrence and development of tumours and killing tumour cells to a certain extent. Overall, the favourable prognosis of tumour patients is closely related to the infiltration of B cells. We have listed various cancer types to prove this hypothesis. Moreover, B-cell density and B-cell activity were significantly increased in tumour patients who responded well to immunotherapy. These results imply that B cells are the key immune cells associated with a favourable tumour prognosis. However, in the practice of translating the immune ability of B cells to tumour immunotherapy, a large number of issues still need to be addressed. First, B cells recognize tumour cell antigens and then act as APCs, which is particularly vital for whether the recognized ones are endogenous or exogenous, which can explore what pathways B cells utilize to enhance the inhibitory effect of immune components on tumours, including endogenous or exogenous antigenic stimuli and related immune signals for induced responses. The relationship between the production of antibodies by PCs and tumours is another hot topic. In addition to enhancing antigen presentation and promoting the killing of other immune cells, the role of different antibody types and their subtypes in different tumours is also worth discussing. It is a potential research direction to explain how B cells respond to tumour cell heterogeneity to achieve the corresponding immune response.

In addition, the relationship between B cells and other immune cells or lymphoid structures is particularly important in different cancer models. In the TME, B cells interact with a variety of immune cells and play a role in tumorigenesis through synergy or antagonism. Among them, B-cell-related cytokines are particularly significant. Core cytokines such as CXCL13, CXCL10 and IL-10 are involved in the recruitment, activation or inhibition of various immune cells. B cells can also reshape the immune microenvironment to provide beds more suitable for T cells to exert their effects and control tumour growth. Of course, these linkages are particularly pronounced in TLSs, where both the degree of B-cell activation and TLS maturation are good indicators of response to ICI therapy. Moreover, some immune complexes have also been found to be associated with inflammatory responses and activation of immune components. In that way, does immune-active individuals or the immune storm after infectious immunization have a positive effect on tumour immunity and its treatment?

Finally, the role of different subsets of B cells in tumours is different in multiple studies. PCs and Bregs are the focus of attention. The functions and phenotypes of various B-cell subsets are complex, and it is necessary to carry out comprehensive marker determination and prognostic analysis of the TME and circulating B cells in various cancers currently relying on single-cell technology. Therefore, in studies of B-cell tumour immunotherapy, it is still necessary to further explore the relationship between its immune mechanisms, changes in the TME with ICI therapy, and the clinical prognosis of various subpopulations. Cancer immunotherapy relying on B cells will undergo a great shift in the near future.

## Data Availability

Not applicable.

## Abbreviations

ICI: Immune checkpoint inhibitor
PD-1: Programmed cell death protein 1
PD-L1: Programmed cell death ligand 1
Bregs: Regulatory B cells
PCs: Plasma cells
CTLA-4: Cytotoxic T lymphocyte–associated protein 4
TME: Tumour microenvironment
TLS: Tertiary lymphoid structure
IL: Interleukin
CD: Cluster of differentiation
scRNA-seq: Single-cell RNA sequencing
CXCL: Chemokine interferon-inducible protein
CCL: Chemokine (C-C motif) ligand
APC: Antigen-presenting cell
MHC: Major histocompatibility complex
DC: Dendric cell
CTL: Cytotoxic T cell
Th: Helper T cell
ADCC: Antibody-dependent cell cytotoxicity
ADCP: Antibody-dependent cellular phagocytosis
ICOS: Inducible costimulatory
Tregs: Regulatory T cells
HCC: Hepatocellular carcinoma
CAR: Chimeric antigen receptor
NSCLC: Non-small cell lung cancer
BC: Breast cancer
CRC: Colorectal cancer
RCC: Renal cell cancer
SCC: Squamous cell carcinoma
GC: Germinal centre
OS: Overall survival
DFS: Disease-free survival
DSS: Disease specific survival
PFS: Progression-free survival
RFS: Recurrence free survival
